# Experimental Verification of Calcite Formation Potential by Ureolytic and Non-Ureolytic Bacterial Strains in Geopolymer Mortar

**DOI:** 10.3390/ma18204795

**Published:** 2025-10-21

**Authors:** Bashar Al Hayo, Orhan Canpolat, Nihal Doğruöz Güngör, Mücteba Uysal, Nahdhoit Ahamada Rachid, Issam Ali

**Affiliations:** 1 Department of Civil Engineering, Faculty of Civil Engineering, Yildiz Technical University, İstanbul 34220, Türkiye; canpolat@yildiz.edu.tr (O.C.); mucteba@yildiz.edu.tr (M.U.); issam.ali@std.yildiz.edu.tr (I.A.); 2 Department of Biology, Faculty of Science, İstanbul University, İstanbul 34134, Türkiye; ndogruoz@istanbul.edu.tr; 3 Institute of Graduate Studies in Sciences, Istanbul University, Vezneciler Fatih, İstanbul 34134, Türkiye; nahamadarachid@gmail.com

**Keywords:** self-healing ability, MICP, injection, water permeability, healing efficiency

## Abstract

This study aimed to examine the calcite precipitation potential of non-ureolytic bacterial strains of two species, *Viridibacillus arenosi* (A_6_) and *Bacillus zhangzhouensis* (D_25_), as compared to the known ureolytic bacterial strain, *Sporosarcina pasteurii* (SP), within geopolymer mortar. Tests were carried out after 56 days of injection treatment to confirm the precipitation process, incorporating healing efficiency measured by ImageJ software, recovery of UPV, water permeability, capillary water absorption, and microstructural and mineralogical analysis SEM/EDS and XRD. The non-ureolytic isolates D_25_ and A_6_ showed the highest healing efficiencies, at 96.9% and 91.9%, respectively, followed by the ureolytic bacteria SP at 77.8%. A_6_ exhibited the most substantial reduction in permeability at 97.3%, indicating extensive crack healing, followed by D_25_ at 92.9% and SP at 82.1%. Furthermore, SEM and EDS analyses confirmed the formation of calcite crystals and calcium depositions in the bacteria-treated samples. Complementary evidence was provided by XRD, which revealed distinct calcium carbonate peaks in the treated specimens, peaks that were entirely absent in the control samples, thus strongly confirming the role of bacterial activity in the precipitation process. The results confirm that non-ureolytic bacteria can efficiently boost calcite precipitation in geopolymer mortars, offering superior healing performance and a more sustainable alternative to ureolytic strains.

## 1. Introduction

Although geopolymer binders are being used as an alternative to ordinary Portland cement (OPC), they are also susceptible to early-age cracks and gaps in internal structures, adversely affecting the structure’s durability [1]. A variety of techniques are available; however, traditional repair systems, including the use of epoxies, latex emulsions, and surface treatments with water repellents like siloxanes or silanes, have limitations such as incompatible interfaces, susceptibility to ultraviolet radiation, unstable molecular structures, high costs, hazardousness, and the emission of toxic gases. Additionally, this technique could be extended to biological healing agents. A possible technique of inducing mineral-producing microorganisms into the concrete is being investigated [2].

Microbial induced calcium carbonate precipitation (MICP) is a common biochemical process in various environments, such as soils, caves, freshwater, and marine sediments. MICP arises from metabolic interactions between diverse microbial communities and organic and/or inorganic compounds in the environment [3]. Different methods have been established for incorporating bacteria into cement-based materials. These methods include directly adding bacterial agents into the mix [4], encapsulating bacteria to preserve their viability until activation upon cracking [5,6], and applying bacteria externally over surface spraying or injection into induced cracks [7].

Unlike traditional concrete, self-healing in geopolymers cannot occur through the hydration of unreacted cement or by the leaching and carbonation of hydration products, as geopolymers do not include anhydrous cement particles or soluble Ca^2+^ ions. Thus, microbial-induced calcium precipitation sealing offers a viable alternative method for enabling self-healing in geopolymer systems [8].

Research on geopolymers has extensively studied their mechanical properties [9,10,11], the generation of high-performance composites, the synthesis of specialized geopolymer mixtures [12,13], and the inclusion of diverse additives to improve mechanical performance [14,15,16]. Nonetheless, implementing bacteria-based self-healing techniques in geopolymers is still largely unexplored, with only a few publications addressing this area. A preliminary method for utilizing bacterial agent-based self-healing geopolymer composites involved formulating a system incorporating the ureolytic bacterium *Sporosarcina pasteurii* into metakaolin-based geopolymer composites [17]. Incorporating bacterial self-healing systems in geopolymer mortars improves durability by reducing permeability and limiting crack propagation. This approach improves material performance, extends the service life of structures, and reduces the maintenance costs [18].

*Sporosarcina pasteurii* is the most studied bacterium for MICP. Other bacteria from Bacillus species are also frequently investigated [19]. *S. pasteurii* is an endospore-forming, alkaliphilic bacterium and is the most used microorganism for biomineralization applications in cement-based materials [20]. Some researchers applied a bacterium isolated from the caves and verified that the mortars with these bacterial additions presented decreased water absorption due to CaCO_3_ precipitation in calcite form [21]. A study on the formation of calcite deposits within caves showed that stalactites and stalagmites within karst caves may result from the presence of microorganisms active in calcite precipitation [22].

Most MICP research, especially in metal remediation, Microbially Enhanced Oil Recovery MEOR, and construction restoration, depends on the ureolysis pathway. However, ureolytic bacteria are not always present or viable in all environments. Certain environmental conditions can inhibit their growth and metabolism, and in some cases, they may fail to survive entirely [23]. Ureolytic bacteria, such as *Bacillus sphaericus*, stimulate CaCO_3_ precipitation by transforming urea into ammonium and carbonate. This process increases pH levels and boosts crystal formation in calcium-rich environments, efficiently sealing cracks.

However, the production of ammonium ions during ureolysis can pose hazards to concrete integrity and the environment, as ammonium may oxidize to nitric acid, dissolving calcium carbonate into calcium nitrate. The possible adverse effects of urease activity should thus be considered [24]. Additionally, ureolytic pathways may be unsuitable for the highly alkaline environments typical of geopolymer matrices, where bacterial survival, activity, and precipitation efficiency may be compromised [25,26]. Conversely, non-ureolytic pathways have gained growing attention as they offer a more sustainable and environmentally friendly alternative, especially under high-pH conditions. Non-ureolytic bacteria do not produce ammonium as a byproduct and are often better adapted to such environments, as their metabolic processes, such as denitrification, can themselves generate sufficient alkalinity to facilitate calcium carbonate precipitation [25,26]. Recent studies have shown that non-ureolytic strains, such as *Priestia aryabhattai* and *Neobacillus drentensis*, can effectively induce calcite precipitation at pH levels ranging from 9–12, conditions relevant to geopolymers [27]. This transition from conventional ureolytic to non-ureolytic MICP thus not only mitigates environmental concerns associated with ammonia production but also aligns with the alkaline conditions required for optimal geopolymer performance.

In this study, two non-ureolytic bacterial strains, *Viridibacillus arenosi* and *Bacillus zhangzhouensis*, isolated from local cave environments, are investigated alongside the well-characterized ureolytic strain *Sporosarcina pasteurii* for their effectiveness in promoting self-healing in geopolymer mortar matrices. The comparative evaluation aims to identify the potential advantages of non-ureolytic pathways for developing sustainable, durable self-healing construction materials.

The paper was organized as follows: Section 2 presents the materials and methods, including details of microbial selection, geopolymer preparation, and testing procedures. Section 3 discusses the results and analysis of self-healing performance and durability. Section 4 provides the conclusions and recommendations for future research.

## 2. Materials and Methods

### 2.1. Section Background

**Figure 1:**
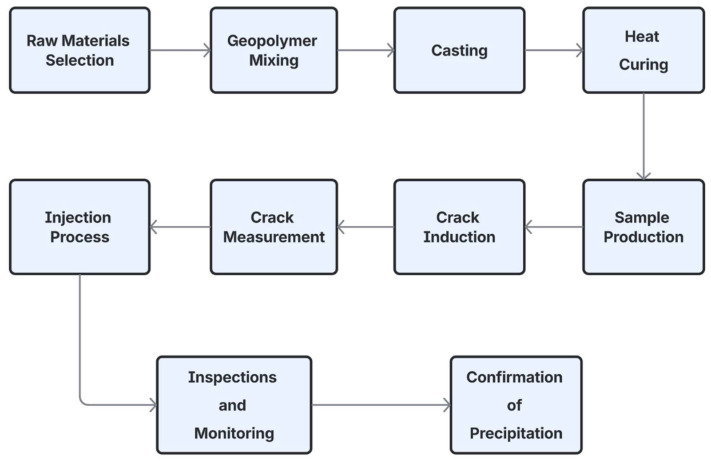
Flowchart of the study stages.

This section details the materials, specimen preparation procedures, experimental methods, and analytical techniques employed in the examination of self-healing efficiency in geopolymer mortars subjected to different bacterial strains. The study employed locally produced waste and byproduct materials as binders, together with techniques for inducing and assessing cracks before and after treatment, adhering to the international standards where applicable. The flowchart, Figure 1, summarizes the stages followed in this study.

### 2.2. Materials

#### 2.2.1. Raw Materials

Ground granulated blast furnace slag (GBFS) and ceramic waste powder (CWP), both obtained from industrial waste in Turkey, are used in this study as the primary binders. GBFS was acquired from a cement plant in Istanbul; CWP was collected as a by-product during the cutting and shaping operations at a ceramic industry. Sodium hydroxide (NaOH, ≥99% purity, white beads) and a commercial potassium silicate solution (K_2_SiO_3_, SiO_2_:K_2_O molar ratio 2:1) were used as alkali activators. Standard sand (BS EN 196-1) was incorporated as the aggregate, and polypropylene fibers were added at 0.5% of the total binder mass to control cracking. The chemical composition of GBFS and CWP was analyzed with X-ray fluorescence (XRF) analysis, as shown in Table 1, and the mix proportions are detailed in Table 2.

#### 2.2.2. Bacterial Strains and Growth Media

Three bacterial strains were used: *Viridibacillus arenosi* (A_6_) and *Bacillus zhangzhouensis* (D_25_), isolated from Dupnisa Cave, Türkiye, their effectiveness was confirmed using samples collected from cave surfaces, and previous findings verified that both local strains could efficiently trigger calcite precipitation under laboratory conditions [28]. Alongside *Sporosarcina pasteurii* (SP, DSM33) which was obtained from the German Collection of Microorganisms and Cell Cultures, this strain is noted for its alkali resistance, calcite precipitation ability, spore production, and non-pathogenic nature [29].

The A_6_ and D_25_ strains were grown on half-strength Tryptic Soy Broth (½ TSB), while SP was grown on ATCC-specific agar. Single colonies were isolated from cultures obtained through the streak plate method to ensure purity and prevent contamination. The selected colonies from these plates were subsequently inoculated in appropriate broth media. All cultures were incubated in a shaker incubator at 120 rpm and 30 °C for 48 h, with colonies isolated for purity, and cell concentrations adjusted to 1.2 × 10^9^ cells/mL for use in experiments. Details of growth media and preparation are presented in Table 3.

### 2.3. Specimen Preparation

Geopolymer mortar samples were prepared with a 50:50 blend of GBFS and CWP as the binder. This binder ratio was selected based on the results of our preliminary experimental program, which included approximately 33 different mix designs. Although previous research has used the same materials with different ratios (e.g., 40% GGBFS and 60% CWP) [30], the final proportions in this study were determined according to our own experimental findings. The alkali activator comprised 12 M NaOH (prepared by dissolving beads in water for 24 h) and K_2_SiO_3_ in a 1:2 ratio. Standard sand and 0.5% polypropylene fibers (by binder weight) were added. Mortar was cast into prismatic molds (4 × 4 × 16 cm) and cylindrical PVC molds (diameter 10.5 cm, height 3.5 cm) to be divided into six groups (3 prismatic and 2 cylindrical shapes for each group). Specimens were demolded after 24 h at 21 °C and 65% relative humidity, cured at 80 °C for 24 h, and thereafter kept under ambient conditions.

**Figure 2:**
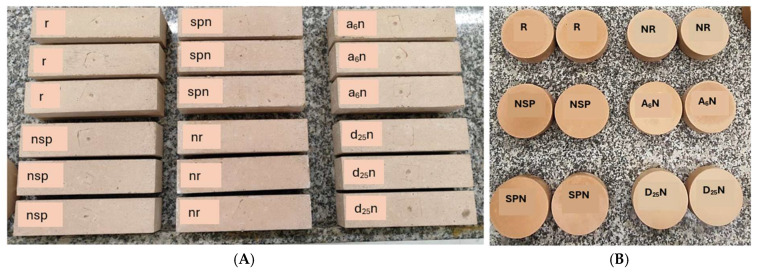
The groups of geopolymer mortar samples used in this study. (**A**) Prismatic Samples; (**B**) Cylindrical samples.

The curing regime of (80 °C, 24 hr.) was chosen based on both the established literature and from our preliminary experiments. This regime is widely stated to accelerate geopolymerization, ensure desirable microstructural properties, and enhance early strength, while minimizing shrinkage and microcracking [30,31]. Table 4 outlines the experimental groups, codes, and injected media; Figure 2 illustrates the prepared specimens, and Table 5 lists the tested specimen types and their respective tests.

### 2.4. Crack Induction and Treatment in Prismatic and Cylindrical Specimens

**Figure 3:**
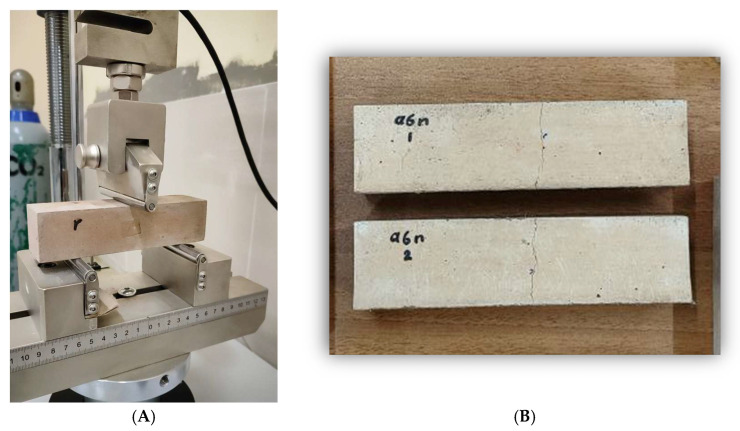
(**A**) Crack creation by loading machine; (**B**) Prisms with created cracks.

**Figure 4:**
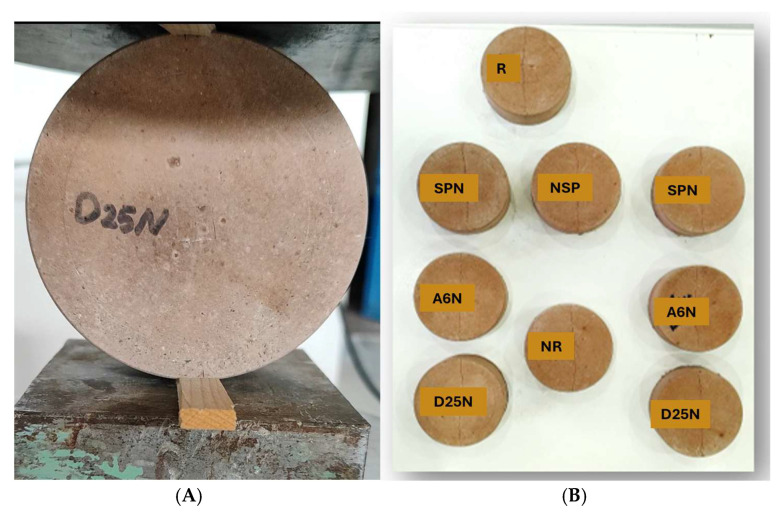
(**A**) crack induced in the specimen with the loading machine; (**B**) Specimens after the creation of the crack.

Cracks in prismatic specimens were induced via three-point bending at a displacement rate of 0.2 mm/min, Figure 3. While cylindrical specimens were cracked using a splitting tensile load (0.05 kN/s) in a Universal Testing Machine (UTM), Figure 4. Polypropylene fibers helped regulate crack width, which was measured at multiple locations along each specimen. Loading stopped once the crack formation became visible, and crack widths ranged from 0.200 mm to 0.260 mm. After crack induction, all specimens received daily injections of different media for 56 days. The evaluation of self-healing performance occurred at 0 and 56 days, representing the baseline (pre-healing) and mature healing stages. This approach was implemented to maintain consistency with previous studies [32,33], which utilized the same time points for bacterial self-healing in cementitious and alkali-activated materials.

### 2.5. Evaluation of Self-Healing Efficiency

#### 2.5.1. Effect of pH on Calcite Precipitation

The ability of A_6_ and D_25_ resins to precipitate calcite in a range of pH levels (8–12) was examined and monitored before their application to geopolymer mortar. The growth of each isolate in each pH value was observed during a maximum of 7 days.

#### 2.5.2. Crack Monitoring and Crack Area Measurement

**Figure 5:**
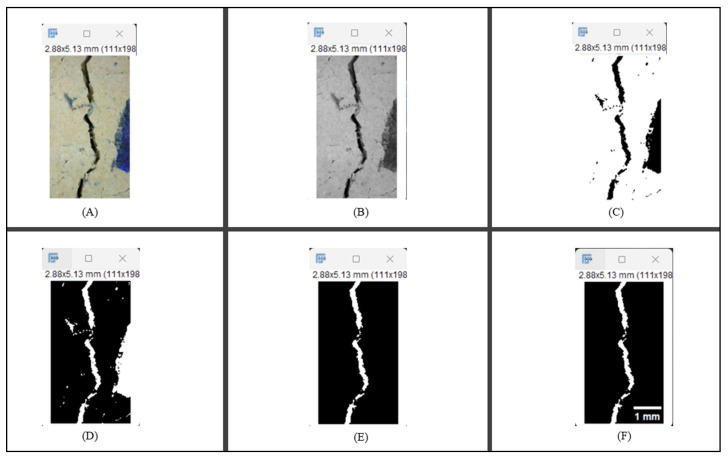
The steps involved in image processing using the ImageJ software. (**A**) Uploaded picture to the software; (**B**) Converted to 8-bit grayscale; (**C**) Threshold adjusted; (**D**) Converted image to binary; (**E**) Binary mask created; (**F**) Reference scale bar placed.

Crack healing was monitored by periodic imaging and analysis using ImageJ software version 2.16.0/1.54p. The sample images were uploaded to the software (Figure 5A). To enhance contrast and distinguish cracks from the background, it was converted to 8-bit grayscale (Figure 5B), thresholded (Figure 5C), and processed to binary (Figure 5D). A binary mask was created for the selected crack region to define its geometry accurately (Figure 5E). Finally, to enable accurate measurement, a reference scale bar was placed within the images (Figure 5F). The crack area was measured at three points along the crack and used to calculate healing efficiency over time, as described by Equation (1). Figure 5 demonstrates the image processing and area calculation workflow.

(1) $$\mathrm{Healing}\;\mathrm{efficiency}\;\left(\%\right)=\frac{A_{0}{–A}_{t}}{A_{0}}\times 100$$

where (A_0_) is the initial crack area before healing and (A_t_) is the crack area at healing time t.

#### 2.5.3. Mechanical and Durability Tests

##### Ultrasonic Pulse Velocity (UPV)

The healing performance was further assessed by the Ultrasonic Pulse Velocity (UPV) test according to ASTM C597-22 [34]; transducers (50 kHz) were applied with a coupling gel, and readings were averaged over duplicate measurements per location. The transducers were placed on opposite faces along the longitudinal axis of the specimen. All measurements were conducted under controlled temperature and humidity.

##### Water Permeability—A Custom-Designed Apparatus

The sealing percentage obtained from the water permeability test is regarded as a significant indicator for evaluating healing efficacy, owing to its strong correlation with the shape and features of the crack. This parameter has been extensively utilized as a primary metric for assessing self-healing efficacy in cementitious-based materials. Generally, there are two methods for conducting water permeability testing: falling head tests and constant head tests [35].

**Figure 6:**
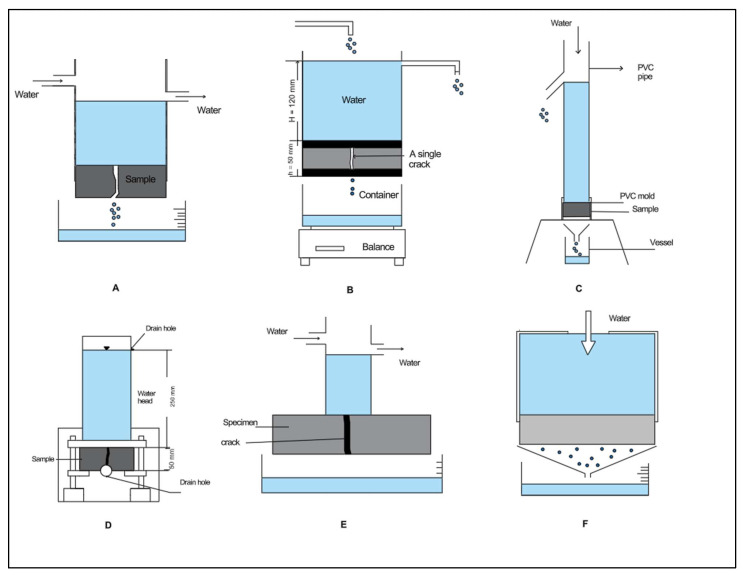
Schematic diagram of the water permeability test apparatus used in the previous studies, adapted from; (**A**) [36], (**B**) [37], (**C**) [38], (**D**) [39], (**E**) [40], (**F**) [41]. All figures were redrawn by the authors.

The water permeability assessment in this study was applied using the constant head method based on Darcy’s law. The methodology is consistent with recent studies such as [36,37]. Other researchers [38,39,40,41] have also used this approach, introducing some modifications to the testing apparatus and applying it to samples of various sizes and shapes. Figure 6 shows a schematic diagram of the apparatus used for water permeability testing in previous studies.

**Figure 7:**
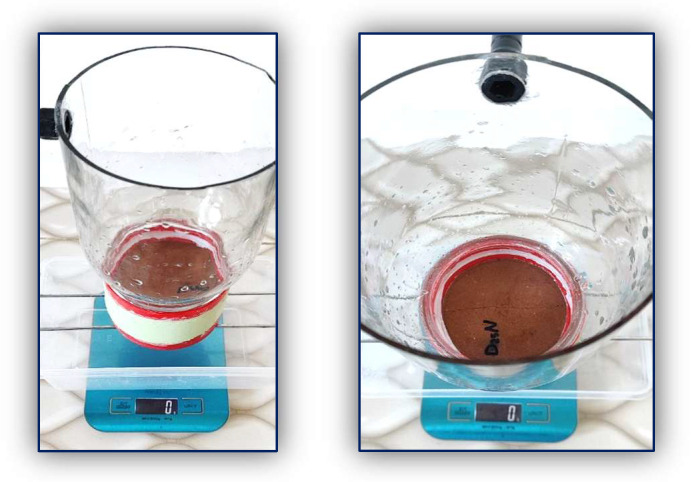
The apparatus used in this study.

**Figure 8:**
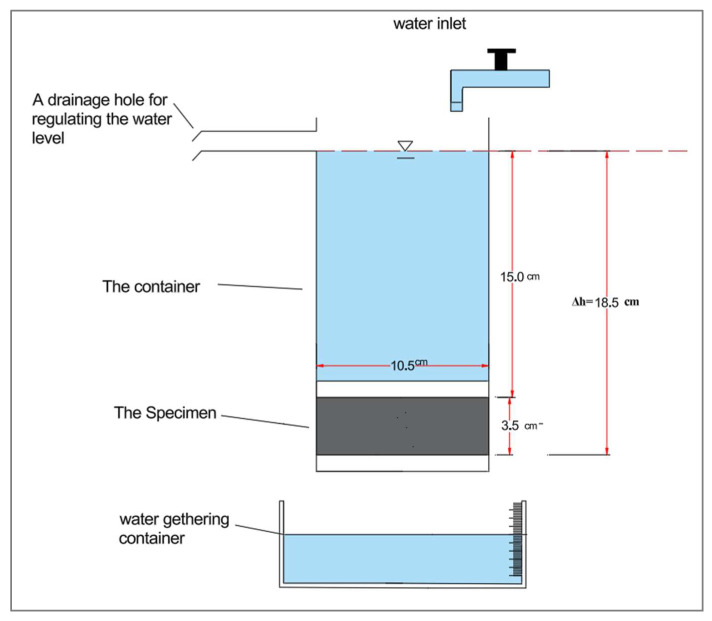
Schematic diagram of the water permeability test.

A custom-designed device was developed to conduct water permeability testing using the constant pressure method in this study based on Darcy’s law. As shown in Figure 7 and Figure 8, the apparatus consists of a glass container held above the specimen during testing by the container’s lid, which is permanently affixed to the surface of each specimen with a strong adhesive. The sides of the specimens are coated with epoxy to prevent any water leakage during the test. The upper container has a top opening for water intake from an external source and a side outlet to keep constant water pressure on the specimen while testing. A separate container is positioned beneath the apparatus to gather water that seeps through the cracks over a designated duration during each test. The volume of water passing through is quantified using a precision balance during the test. All tests were performed at 23 ± 2 °C and 55–65% RH. Each group’s results represent the average of two measurements.

Water permeability rate through cracks was determined using the volumetric flow rate and specimen geometry, following Equation (2), derived from Darcy’s law and widely applied for evaluating permeability in cementitious materials.

(2) $$k=\frac{Q\cdot L}{A\cdot \Delta h\cdot t}\times \;100$$

where:

k: Permeability coefficient (m/s).

Q: Water flowing within the cracks during the established time (m^3^).

L: Specimen Length (m).

A: The cross-sectional area of the specimen (m^2^).

Δh: The difference of hydraulic pressure (m).

t: Time of the test (s).

##### Capillary Water Absorption

Capillary water absorption tests were performed at the end of the bacterial treatment period, in accordance with ASTM C1585-20 [42]. Cylindrical specimens were subjected to oven drying at 50 ± 5 °C until a constant mass was attained. After cooling to room temperature, the lateral surfaces of each specimen were sealed with an impermeable coating, leaving only the bottom surface of the specimen exposed to water. Then, the specimens were placed on supports in shallow trays, with the exposed surface in contact with water to a depth of 2–3 mm. Water uptake was recorded at consistent intervals: 1, 5, 10, 20, 30, 60, 120, 240, 480, 720, and 1440 min (up to 24 h) after initial contact.

##### Microstructural Characterization

Following the healing process, selected specimens were analyzed by scanning electron microscopy (SEM), energy-dispersive spectroscopy (EDS), as well as X-ray diffraction (XRD) to ascertain the mineralogical composition of the precipitates and confirm the presence of calcite.

##### SEM/EDS Analysis

SEM and EDS analyses were performed with a Zeiss EVO LS 10 SEM microscope integrated with an EDAX Elemental EDS detector (Oberkochen, Germany). During SEM imaging, the accelerating voltage was established within the range of 5–20 kV and was accurately adjusted according to the conductivity properties of the specimens. Non-conductive specimens were coated with a thin gold–palladium film under high vacuum to minimize charging. Mortar samples were cut into pieces of approximately (1 × 1 cm), cleaned with distilled water, and oven-dried at 60 °C for 24 h prior to analysis.

##### X-Ray Diffraction (XRD) Analysis

XRD analysis was performed using a Malvern PANalytical X’Pert PRO diffractometer (Malvern PANalytical, Almelo, The Netherlands) equipped with a Cu Kα radiation as the source (Kβ = 1.39225 Å, Kα1 = 1.54060 Å, Kα2 = 1.54443 Å) under operating conditions of 40 kV and 45 mA. The scanning settings involved a 2θ range of 5.00–99.99°, with step size of 0.02°. For sample preparation, the powders were finely ground and affixed to a flat holder, then pressed smoothly to ensure a flat surface.

## 3. Results and Discussions

### 3.1. General Overview

This section presents the main findings on the self-healing and durability of geopolymer mortars. Results are organized by pH effects on calcite precipitation, crack monitoring, mechanical and durability tests (ultrasonic pulse velocity, water permeability, capillary absorption), and microstructural analyses (SEM/EDS, XRD). Each subsection discusses the main observations and their implications for bacterial self-healing efficiency. To ensure clarity and consistency, we present and discuss the results step by step in the same order as the preceding section.

### 3.2. Effect of pH on Precipitation

**Figure 9:**
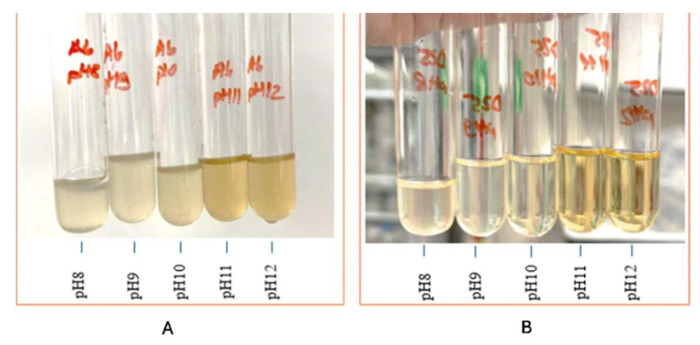
Examination of the ability of calcite precipitation at a range of pH levels. (**A**) *Viridibacillus arenosi* (A_6_), (**B**) *Bacillus zhangzhouensis* (D_25_).

**Figure 10:**
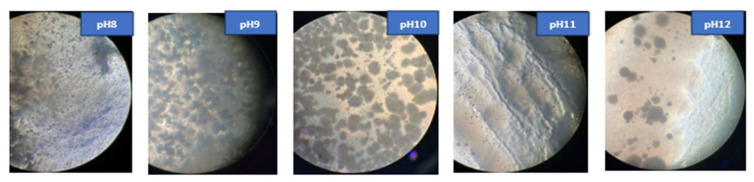
Growth of *Viridibacillus arenosi* (A_6_) and crystal formation in B4 medium under light microscopy ×40.

**Figure 11:**
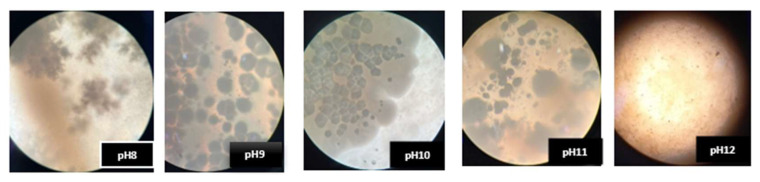
Growth of *Bacillus zhangzhouensis* (D_25_*)* and crystal formation in B4 medium under light microscopy ×40.

The ability of cave isolate samples A_6_ and D_25_ to promote crystal formation and precipitation in B4 medium across different pH levels (8–12) was demonstrated, as shown in Figure 9. Their development and crystallization were observed at each pH level for up to 7 days. Moreover, the morphological characteristics of the crystals developed under these varying environments are illustrated in Figure 10 and Figure 11, which display representative microscopic observations acquired using a light microscope (NIKON E100, ×40 magnification), manufactured by Nikon Corporation, headquartered in Shinagawa, Tokyo, Japan.

The results indicated that the isolate A_6_ demonstrated considerable precipitation, especially at pH 9–11. D_25_, on the other hand, produced minimal precipitation at pH 8–9 and moderate precipitation at pH 10. This is consistent with the research achieved by Shaheen et al. (2021) [43], who reported that non-ureolytic bacteria achieve maximal calcite precipitation at pH 10, using carbon sources via respiration to produce carbonate ions under alkaline conditions.

A study by Zheng Y et al. (2025) [44] demonstrated that the urease enzymatic activity in *Sporosarcina pasteurii* is optimal at pH 7–8 and drastically diminishes at pH 11, indicating that highly alkaline environments may adversely affect the calcite precipitation efficiency of these bacteria. On the other hand, Harnpicharnchai P et al. (2022) [27] looked at non-ureolytic bacterial species, *Priestia megaterium* and *Neobacillus drentensis*, and found that they could effectively cause calcium carbonate precipitation in a pH range of 9–12, making them suitable for use in highly alkaline conditions.

### 3.3. Crack Monitoring and Crack Area Measurement

**Figure 12:**
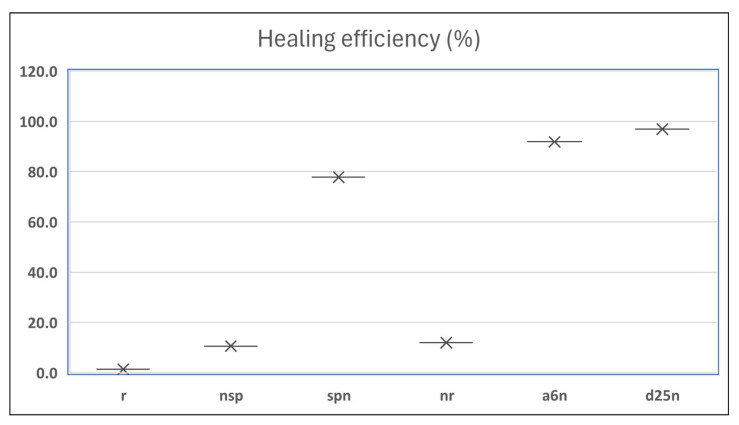
Healing efficiency by measuring the crack area.

The images taken before and after the injection process, evaluated using ImageJ software, revealed significant differences in the efficiency of crack closure. These disparities are demonstrated in the photographs in Table 6, where the sealing rate was precisely measured and recorded by comparing the initial and final pictures after the treatment period, as presented in Table 7 and Figure 12. D_25_N reached the greatest crack filling rate, with a closure rate of 96.9%. A_6_N had a closure rate of around 91.9%, while SPN produced a precipitation rate of 77.8%.

On the other hand, reference samples, which were not treated with any solution, showed no noticeable change during the treatment period. At the same time, the observations from the nutrient-only injected specimens suggest that non-biological factors may contribute to a partial visual crack closure of 10.6% with the NSP specimen and 12% with the NR specimen.

The phenomenon is mainly ascribed to the presence of fibers in the mix, which serve to bridge cracks and provide efficient nucleation sites primarily in the presence of calcium-rich nutrients. This explanation is supported by the findings of Mahmoodi et al. [45], who demonstrated that the formation of healing products mainly occurs at the nucleation sites formed by the distribution of PVA fibers within the cracks, gradually filling and bridging the cracks.

In the study, non-ureolytic Bacillus species (*B. pseudofirmus*, *B. cohnii*, *B. halodurans*) were used in self-healing concrete. These types showed the ability to survive in cementitious matrices and efficiently seal microcracks up to nearly 0.13 mm via calcite precipitation. *B. pseudofirmus* demonstrated the highest healing efficiency, achieving approximately 50% crack closure within 56 days [6].

Further research utilized a co-culture of two bacteria, ureolytic *Sporosarcina pasteurii* and non-ureolytic Bacillus thuringiensis, which significantly accelerated crack healing by enhancing bacterial growth and promoting rapid CaCO_3_ nucleation. The accelerated growth rate of the non-ureolytic organism *B. thuringiensis*, unlike the ureolytic bacteria, resulted in more rapid respiration that produced CO_2_, which declines pH and creates nucleation sites, thus promoting CaCO_3_ precipitation [46].

### 3.4. Ultrasonic Pulse Velocity

**Figure 13:**
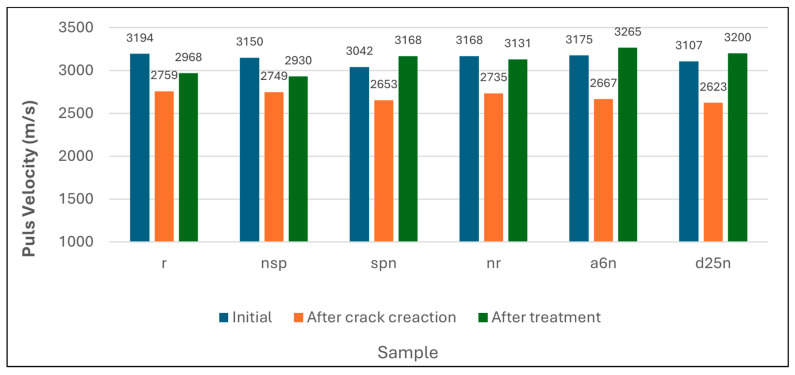
Initial, After Crack Creation, and After Treatment of the UPV test.

UPV testing was conducted on prismatic geopolymer mortar samples. UPV measurements were taken at different specific stages: before crack formation, immediately after induced cracking, and after 56 days of healing, as shown in Figure 13.

A reduction in UPV was noted in all specimens upon crack formation, which aligns with the predicted loss of material integrity. This behavior is consistent with the studies of Tinoco and Pinto (2021) [47], who reported that cracks disrupt ultrasonic wave propagation due to increased scattering, resulting in prolonged arrival times and reduced stiffness in reinforced concrete beams.

All samples exhibited UPV recovery after 56 days. Control samples showed a slight recovery of close to 7.6%, probably due to slow ongoing densification via delayed geopolymeric reactions, consistent with the observations of Kumar et al. (2024) [48] in geopolymer-based systems. Whereas, specimens treated with both nutrients and calcium lactate alone (NSP and NR) also showed increases in UPV, with increases of 6.6% and 14.5%, respectively. Such results may reflect partial chemical precipitation, though it is less effective than biological processes.

An enhancement in UPV was observed in specimens injected with bacteria and calcium lactate across all three bacterial strains (SPN, A_6_N, and D_25_N), where UPV values exceeded their original pre-cracking levels. An increase was attained by 19.4%, 22.4%, and 22%, respectively, indicating enhanced structural integrity. This outstanding effect is attributed to microbial calcium carbonate precipitation (MICP), creating crystalline bridges and filling internal voids.

**Figure 14:**
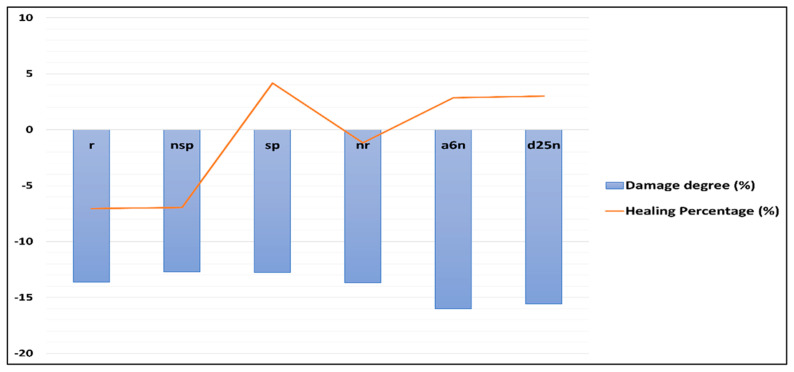
The damage and healing percentage of geopolymer mortar specimens.

Figure 14 illustrates the degree of damage resulting from cracking and the associated self-healing ratios after treatment in geopolymer mortar specimens. The data demonstrate that samples with more damage typically exhibited higher self-healing ratios, especially those subjected to bacterial treatment. This trend was steady across all three types of bacterial treatment, showing superior healing performance relative to specimens treated without bacteria and untreated control samples. These observations align with findings reported in previous studies [49], which examined the relationship between damage severity and the efficacy of self-healing mechanisms in mortar. Tanyildizi et al. reviewed the application of *Sporosarcina pasteurii* bacteria to heal metakaolin-based geopolymer mortars through three techniques: submersion, injection, and spraying. The injection approach exhibited the most effective self-healing performance [49]. Singh and Gupta [50] examined the self-healing properties of samples with UPV measurements. Their findings indicated that the self-healing rate increased in direct correlation with the level of damage across all mixtures.

### 3.5. Water Permeability

Water permeability was evaluated using the constant head pressure on geopolymer mortar specimens with artificially induced cracks, as described in the methodology section, to assess the healing potential after treatment. The permeability coefficient (k) was calculated by applying Darcy’s Law. The water permeability of cracked specimens is closely linked to the extent of crack healing. Thus, the effectiveness of the self-healing process can be quantitatively assessed using the impermeability ratio as an indicator [40].

**Figure 15:**
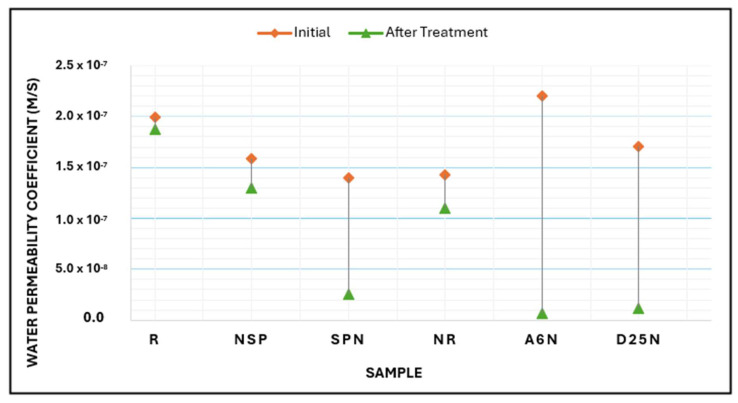
Water Permeability Coefficient.

Table 8 and Figure 15 illustrate the results, which present the permeability coefficients of all specimens before and after treatment. The test time was constant for all samples, set at 5 min (300 s). The initial permeability coefficient values were recorded between 1.4 × 10^−7^ and 2.21 × 10^−7^ m/s.

Compared to the other groups, the samples exposed to bacterial treatment presented a reduction in the permeability coefficient. The specimen treated with A_6_N showed a 97.3% decrease in permeability, indicating that most of the crack is sealed. Whereas D_25_N and SPN treatments reduced permeability by 92.9% and 82.1%, respectively.

The nutrient-only specimens (NSP and NR) demonstrated a partial decrease of 18.4% and 23.1%, respectively, indicating a limited healing effect that may be due to residual or indigenous microbial activity or chemical interactions within the matrix. The observed slight reduction may result from non-biological chemical precipitation. In high pH environments, such as those found in alkali-activated materials, calcium ions from calcium lactate can spontaneously precipitate when carbonate ions or dissolved CO_2_ are present. This reaction, however, is unregulated and often superficial. Wiktor and Jonkers (2011) [51] present similar interpretations, suggesting that certain chemical sealing can occur in the absence of bacterial resins, although it may lack structural depth and durability. The control specimens demonstrated negligible alteration at 5.5%, indicating a lack of self-healing activity without biological or chemical intervention.

The decrease in permeability observed in bacteria-treated specimens is due to the biogenic precipitation of calcium carbonate (CaCO_3_), which sealed the cracks and reduced the connected porosity. This phenomenon corresponds with the findings of Rong et al. (2020) [40], who examined the impact of bacterial concentration on the healing efficiency of cement-based materials. They treated cracks of 200–300 μm width using *Bacillus pasteurii* with varying concentrations. The specimens treated with 10^9^ CFU/mL exhibited nearly complete recovery of permeability. Research on permeability revealed that specimens containing bacteria show water permeability values up to ten times lower than those lacking bacterial content [52]. Further studies have also reported that including bacteria improves permeability characteristics [53]. These results are consistent with the present investigation, which similarly observed significant self-healing performance in geopolymer mortar samples treated with bacterial injections.

### 3.6. Capillary Water Absorption

**Figure 16:**
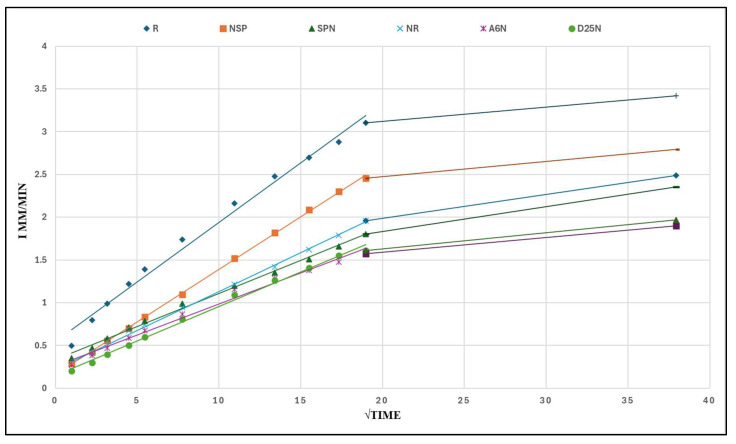
Capillary absorption versus square root of time for specimens.

Capillary absorption tests were conducted according to ASTM C1585. As shown in Figure 16, cumulative water absorption per unit area was recorded at certain time intervals and plotted versus the square root of time. For each group, the initial and secondary sorptivity slopes were determined as the best-fit lines for the periods of 0–6 h and 6–24 h, respectively.

**Figure 17:**
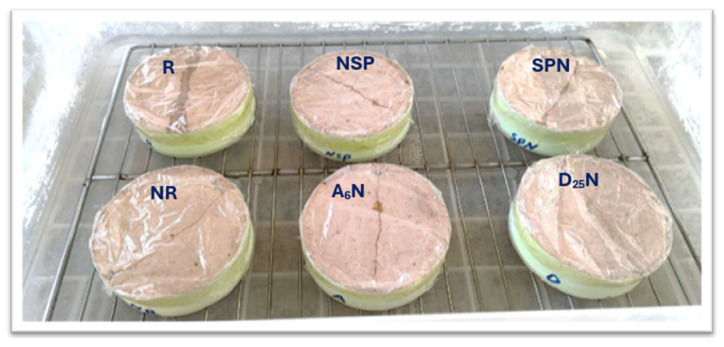
Capillary Water Absorption Test.

**Figure 18:**
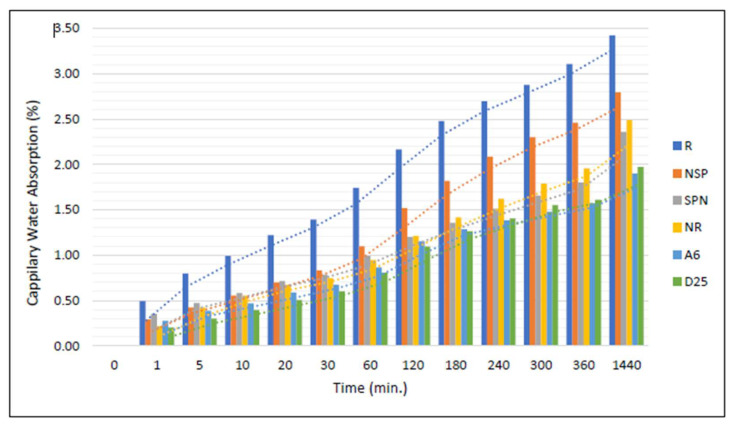
Capillary Water Absorption of samples.

On the other hand, Figure 17, measuring water uptake by capillarity over time, from initial contact up to 1440 min (24 h), on cylindrical geopolymer mortar specimens. The evolution of absorption percentage is detailed in Table 9 and Figure 18. The control specimen R exhibited the highest absorption, serving as the baseline. All treated specimens showed lower absorption, indicating improved resistance to capillary uptake.

The most significant reductions were seen in specimens A_6_N and D_25_N, with approximately 55–57% of the control absorptions, followed by NSP and NR. These trends confirm the positive impact of bacterial treatment, particularly for samples injected with bacterial strains and calcium lactate, as the decrease in capillary absorption is consistent with permeability reduction findings. These results are consistent with observations in Ali et al. (2025) [54] were bacterial treatments significantly reduced water absorption and capillary entry.

### 3.7. SEM/EDS Analysis

**Figure 19:**
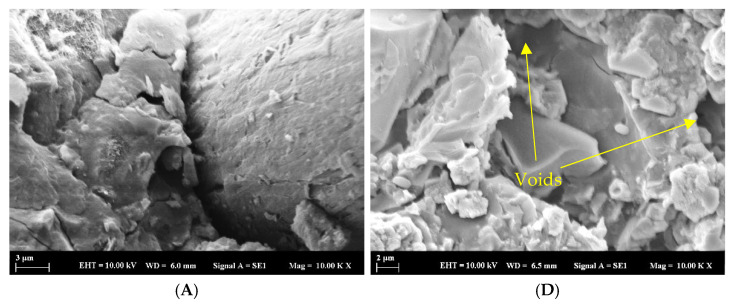

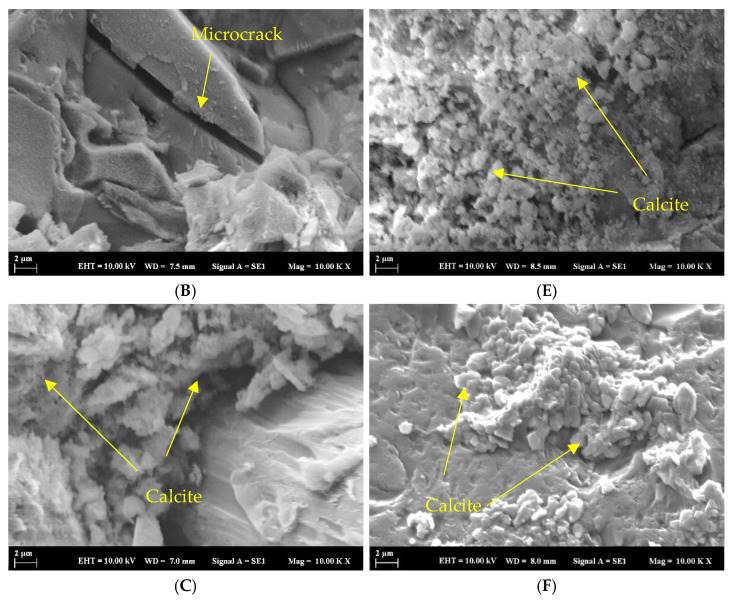
SEM images of the specimens at 10,000× magnifications after treatments by different bacterial types, showing the surface morphology and bacterial calcite deposition at the crack area. R (**A**), NSP (**B**), SPN (**C**), NR (**D**), A_6_N (**E**), D_25_N (**F**).

Figure 19 illustrates the scanning electron microscopy (SEM) observations of the crack regions in geopolymer mortar specimens subjected to three distinct bacterial species. The analysis interprets the morphology of the mineral deposits developed within the cracks. SEM imaging at magnifications 10,000× was performed on all sample groups, which included control, nutrient-only, and bacteria-treated specimens.

According to Oral and Ercan, sedimentation-induced calcites commonly exhibit a range of morphologies, such as cubic structures, flower-like formations, or irregular shapes [55]. It can be noted that the control sample (Figure 19A) showed no visible calcium carbonate precipitations when examined by SEM. There was a complete absence of any secondary crystalline or mineral formations resulting from biological or chemical treatment. However, partial surface accumulations were observed in the samples treated with nutrients and lactate. Samples (Figure 19B,D) showed precipitations associated with calcium precipitation, which may have originated from the lactate solution injected with the nutrients. These precipitations were also observed within the polymerization products of the main mortar components, namely slag and ceramic powder. The examined precipitations are likely attributed to the reaction products of the geopolymer mortar, rather than bacterial activity.

In contrast, bacteria-treated specimens (Figure 19C,E,F) exhibited clear crystalline deposits with distinct morphologies ranging from cubic to irregular forms, indicative of calcite precipitation resulting from microbial activity. Their spatial distribution and density were more obvious than the other groups, offering clear visual confirmation of precipitation processes resulting from the bacterial agent following the treatment process. It was also observed that the polypropylene fibers within the crack regions of the bacteria-treated samples showed a distinct brightness under SEM imaging, unlike the control samples. This phenomenon is linked to the accumulation of mineral deposits, indicating that the fibers acted as effective nucleation sites for calcium carbonate precipitation. The findings align with earlier research, such as that reported by Zhang L et al. [56], which demonstrated that fibers can play a comparable role in promoting mineral formation by serving as nucleation substrates.

**Figure 20:**
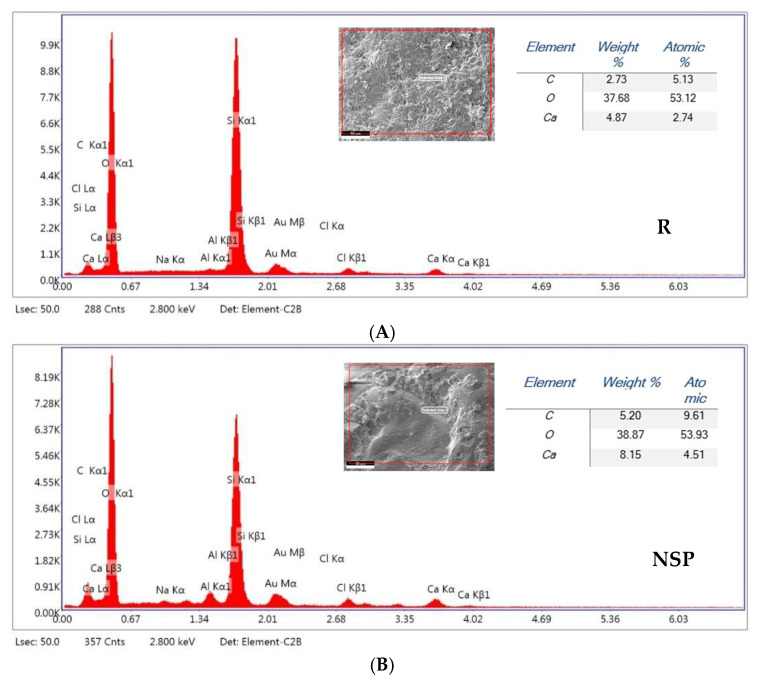

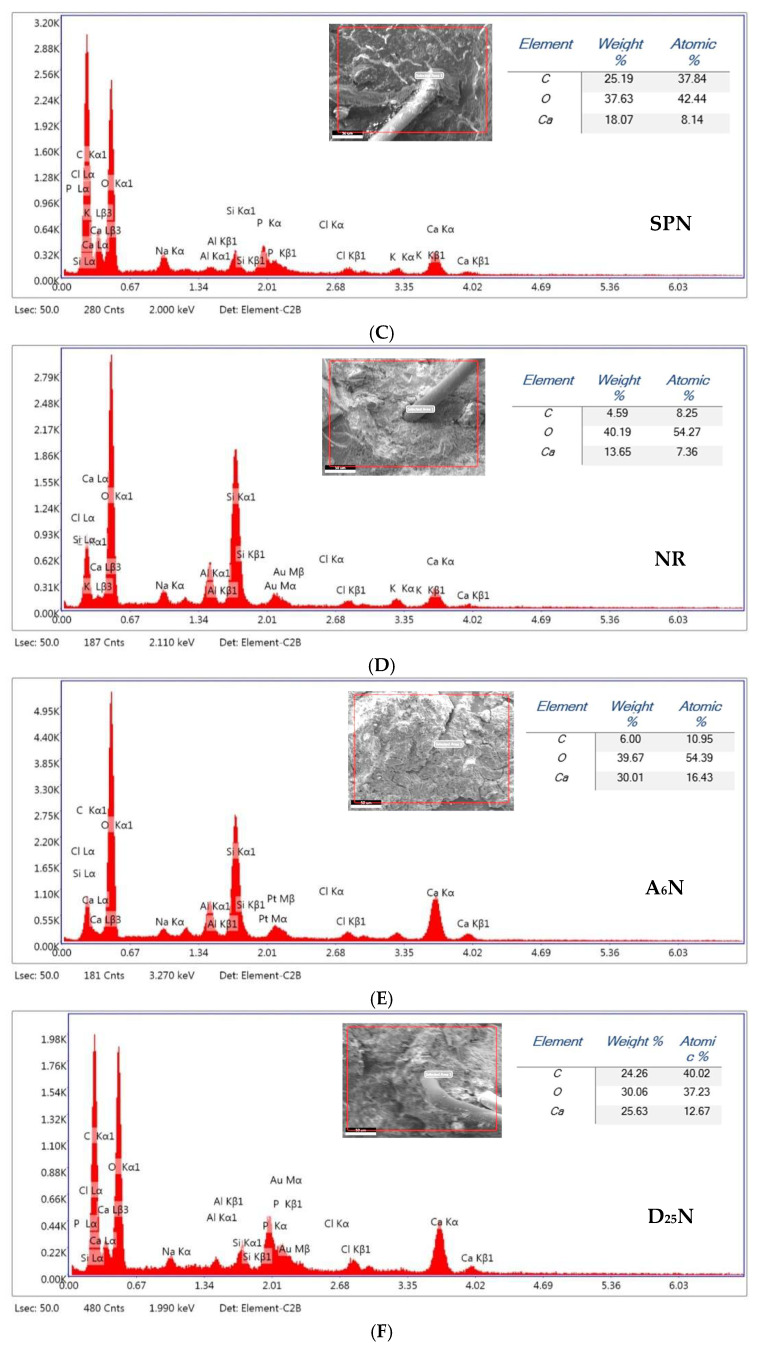
EDS spectrum patterns and element contents of all specimens, including weight and atomic percentages. R (**A**), NSP (**B**), SPN (**C**), NR (**D**), A_6_N (**E**), D_25_N (**F**).

Energy-dispersive X-ray spectroscopy (EDS) revealed that calcium (Ca), oxygen (O), and carbon (C), elements associated with calcium carbonate precipitation, were present in all samples examined. The self-healing compounds formed within the cracks, primarily composed of these three main elements (Ca, O, and C), indicate that calcium carbonate is the predominant self-healing product. This finding was confirmed by Zhang et al. [56]. (Figure 20A–F) reveals some variations in relative and atomic weights between the different groups. The calcium detected in the control group (Figure 20A) may be due to the original raw materials within the mortar mixture, primarily ceramic powder and ground granulated blast furnace slag (GGBS), both of which are known to contain calcium phases. This observation is consistent with the findings reported by Nguyễn et al. [57]. However, samples injected with nutrient media, such as TSB and ATCC, aligned with calcium lactate (Figure 20B,D) and showed moderate calcium quantities. This would potentially be due to abiotic precipitation triggered by the presence of a calcium source under appropriate environmental conditions or due to residual ions binding to the pore matrix. Similar studies have discussed these findings, demonstrating that non-biological pathways also contribute to mineral deposition in the presence of calcium-rich precursors [58].

On the other hand, compared to the different groups, an increase in calcium content was observed in the specimens treated with bacterial solutions in conjunction with calcium lactate, especially in (Figure 20E,F), with 30% and 25.6%, respectively, suggesting biologically induced carbonate precipitation (MICP). Geomicrobiological evidence indicates that the isolated cave bacterium, *Bacillus zhangzhouensis* D_25_, studied by Türkgenci et al. [28], and used in this study, may contribute to calcite precipitation in cement-based systems. The primary compositions of the biodeposits are like pure calcium carbonate (CaCO_3_) crystals, as reported by Seifan et al. [59], indicating that the resulting particles are produced by bacteria. Meanwhile, a quantity of silicon (Si) and aluminum (Al) was detected in the samples, which is likely attributed to the formation of calcium-alumina-silicate-hydrate (C-A-S-H), a typical hydration product of alkali-activated slag.

### 3.8. X-Ray Diffraction (XRD) Analysis

The XRD analysis exposed the presence of quartz (Q), aluminosilicate (S.A), and CaCO_3_ (calcite) phases in the tested specimens. Match software, version 3.16 Build 283, was used to identify the phases according to COD (the crystallography open database) as a source of entry.

**Figure 21:**
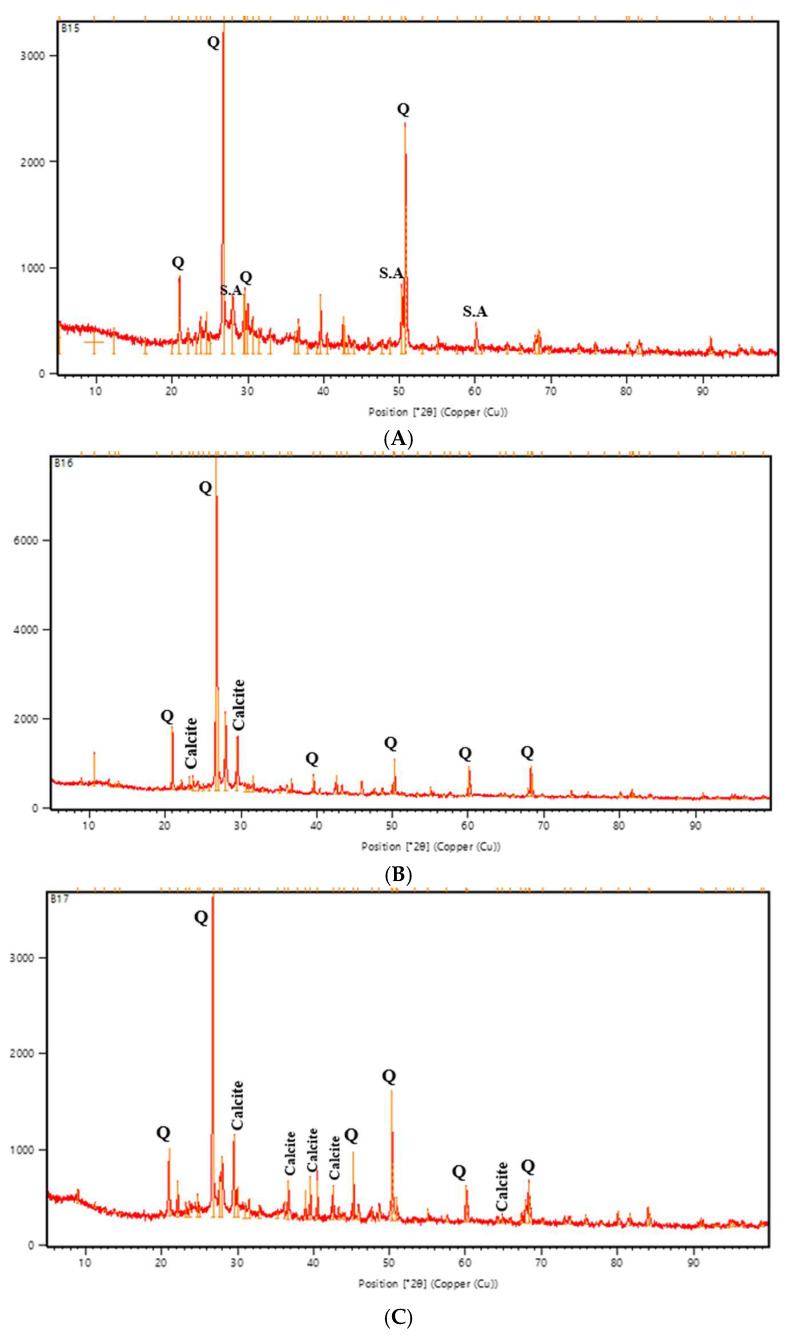

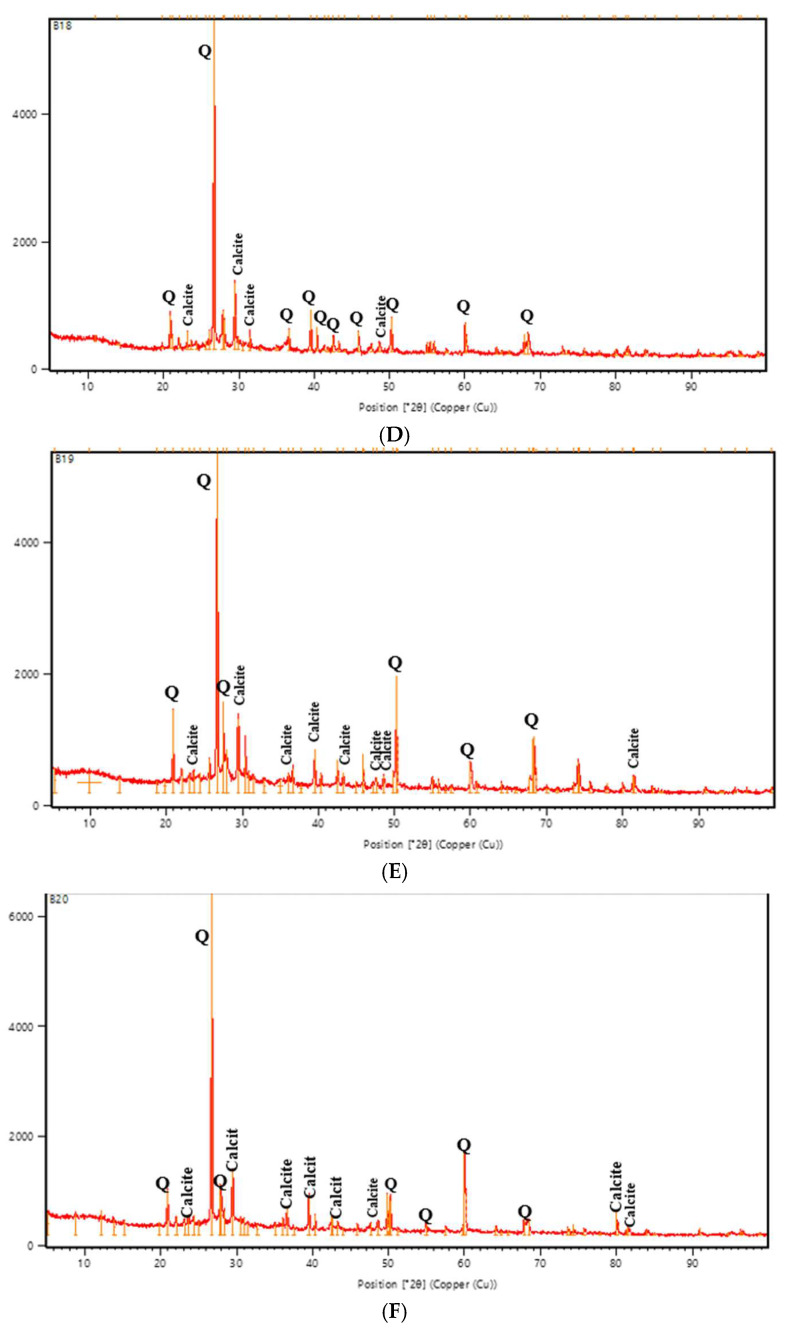
XRD patterns in the mouths of the cracks for all groups. Q: Quartz, S.A: Aluminosilicate, Calcite: CaCO_3_. The red line represents the experimental XRD pattern, while the orange line corresponds to the reference diffraction peaks obtained from the COD database. R (**A**), NSP (**B**), SPN (**C**), NR (**D**), A_6_N (**E**), D_25_N (**F**).

The quartz phase, commonly noticed in geopolymer-based materials due to the use of ceramic powder and slag as precursor substances, is recognized through characteristic peaks at approximately 20.8°, 26.6°, and 50.1° 2θ, which match with the entry number (PDF# 96-500-0036). These reflections were observed in all specimens, as expected given the nature of the aluminosilicate-based matrix (Figure 21A–F). In addition, calcite peaks were identified in the bacterial-treated samples (Figure 21C,E,F), with diffraction angles appearing at approximately 29.4°, 39.4°, and 43.1° 2θ. These peaks match the (104), (110), and (113) crystallographic planes of calcite and align with the standard reference using the entry number (PDF# 96-901-6707). Several studies, including [60,61,62], recognized these entry numbers for calcite and quartz. These samples’ calcite presence validates the occurrence of microbially induced precipitation as a component of the crack-healing mechanism.

In contrast, no calcite peaks were detected in the control specimens (Figure 21A), showing only (Q) peaks and some peaks attributed to secondary (S.A) phases. Minor or weak calcite peaks appeared in the nutrient-treated specimens without bacterial inoculation, represented by Figure 21B,D, suggesting limited non-biological precipitation. Meanwhile, the minor calcite peaks presented in nutrient-only samples can be attributed to spontaneous chemical interactions between calcium lactate and dissolved CO_2_ in the highly alkaline geopolymer circumstance, as discussed by Bernal et al. (2021) [63]. Okyay and Rodrigues support this hypothesis. They discovered that calcite precipitation is not limited to biological activity alone but can also be induced by chemical (abiotic) conditions such as high pH and urea, as some precipitation was observed even in the absence of active bacterial activity [22].

## 4. Conclusions

This study showed that bacterial treatment, particularly with non-ureolytic strains (D_25_N and A_6_N), led to the highest healing efficiencies in geopolymer mortars, surpassing both the ureolytic strain and control specimens. This highlights the strong potential of non-ureolytic pathways for effective crack closure in alkaline environments.

Bacterial samples also exhibited clear improvements in UPV, significant reductions in permeability and capillary water absorption, and microstructural evidence of calcite precipitation. These findings confirm that bacterial-induced healing not only cracks seals but also improves the durability and structural integrity of geopolymer mortars.

Overall, the results emphasize the promise of non-ureolytic bacteria for evolving sustainable, self-healing construction materials.

Future research should consider the self-healing performance and long-term durability of geopolymer mortars under different environmental conditions, including freeze–thaw cycles and aggressive chemical environments. Further research into the possible self-healing and sustainability of non-ureolytic strains is also essential, focusing on their distinct calcite precipitation mechanisms and comparative environmental benefits. Furthermore, systematic assessment of the impact of local pH changes induced by bacterial activity on the aluminosilicate framework and the long-term mechanical stability of geopolymer mortar would be valuable.

## Figures and Tables

**Table 1:** The main chemical compositions of GGBS and CWP.

Oxide	Percentage (%)	
	GGBS	CWP
SiO_2_	35.92	60.42
Al_2_O_3_	9.02	16.00
CaO	53.06	15.38
Fe_2_O_3_	1.99	8.20

**Table 2:** Mix proportions of geopolymer mortar in terms of binder weight.

Binder	GGBS	CWP	Sand	Activators		Fiber
				NaOH	K_2_SiO_3_	
1	0.5	0.5	2.5	0.22	0.43	0.005

**Table 3:** Culture Media Used for Preparation of Bacterial Strains.

Medium Type	Main Components/1 L	Bacterial Isolates Codes	Notes
ATCC Medium 1376	Tris’s base: 6.05 g, (NH_4_)_2_SO_4_: 10.0 g, Yeast extract: 1 g, Glucose: 1 g, Urea: 20 g, pH: 9	SP	
½ Tryptic Soy Agar (½ TSA)	Tryptone: 8.5 g, Soy peptone: 2.5 g, NaCl: 2.5 g, Agar: 15 g, pH: 7.3	A_6_ & D_25_	Autoclave at 121 °C for 15 min
½ Tryptic Soy Broth (½ TSB)	Tryptone: 8.5 g, Soy peptone: 2.5 g, NaCl: 2.5 g, pH: 7.3	A_6_ & D_25_	Liquid medium; Autoclave at 121 °C for 15 min
Calcium Lactate Solution	Calcium lactate: 20 g, Distilled water: 1000 g	For all strains	Dissolved, autoclaved, and used as a mineralization stimulant

**Table 4:** Sample codes and injected mediums.

Group No.	Sample Code	Injected Medium
1	r, R	Reference samples without any injection process
2	nsp, NSP	ATCC + calcium lactate
3	spn, SPN	SP bacteria + calcium lactate
4	nr, NR	1/2TSB + calcium lactate
5	a_6_n, A_6_N	A_6_ bacteria + calcium lactate
6	d_25_n, D_25_N	D_25_ bacteria + calcium lactate

(Sp) *Sporosarcina pasteurii*, (A_6_) *Viridibacillus arenosi*, (D_25_) *Bacillus zhangzhouensis*, (ATCC) Medium 1376, provides optimal conditions for urease activity for SP. (1/2TSB) diluted Tryptic Soy Broth used to support the growth and metabolic activity of A_6_ and D_25_ bacteria species in this study.

**Table 5:** Type of samples and tests carried out for all series.

Sample Shape	Group	Sample Code	Method of Creating Cracks in Specimens	Treatment Method After Crack Creating	Investigation of Healing Efficiency
Prisms (4 × 4 × 16) cm	1	r	cracks were made by applying load at a displacement speed rate of 0.2 mm/min.	21 °C and RH ≥ 65	Crack Monitoring, Crack Area measurement, and UPV (Recovery)
	2	nsp		Injection with different mediums as detailed in Table 4	
	3	spn			
	4	nr			
	5	a_6_n			
	6	d_25_n			
Cylinders (3.5 h, 10.5 Ø) cm	1	R	Single cracks were made by splitting tensile load at load rate of 0.05 KN/s.	21 °C and RH ≥ 65	Healing efficiency, water permeability, and capillary water absorption tests
	2	NSP		Injection with different mediums as detailed in Table 4	
	3	SPN			
	4	NR			
	5	A_6_N			
	6	D_25_N			

**Table 6:** Crack Monitoring for all groups.

Group	Before Treatment	After Treatment	Processed by ImageJ Software	
			Before Treatment	After Treatment
R	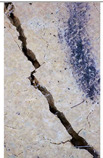	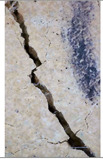	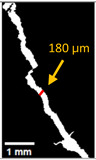	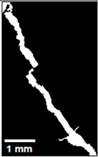
NSP	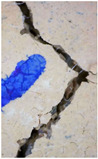	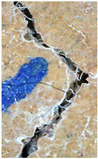	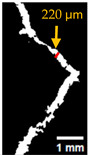	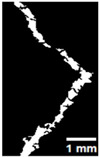
SPN	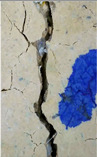	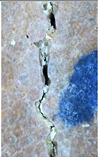	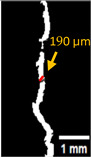	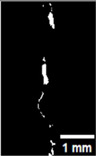
NR	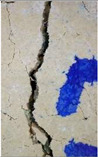	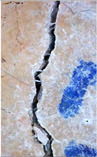	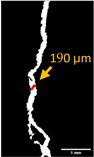	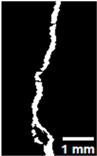
A_6_N	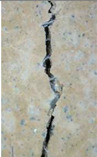	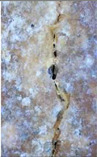	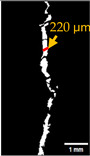	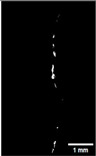
D_25_N	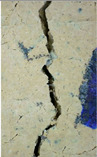	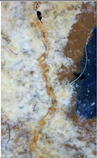	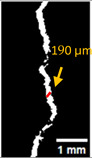	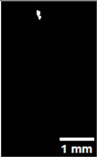

**Table 7:** Area of cracks and healing efficiency.

Sample	Area of Crack (mm^2^)		Healing Efficiency (%)
	Before Treatment	After Treatment	
r	1.399	1.380	1.4
nsp	1.718	1.536	10.6
spn	0.731	0.162	77.8
nr	1.035	0.911	12.0
a_6_n	0.85	0.069	91.9
d_25_n	0.811	0.025	96.9

**Table 8:** Permeability Coefficient of Specimens.

Specimen	Average Crack Width (µm)	Permeability Coefficient K (m/s) × 10^−7^	
		Initial	After Treatment
R	230	2.0	1.9
NSP	247	1.6	1.3
SPN	200	1.4	0.3
NR	227	1.4	1.1
A_6_N	203	2.2	0.1
D_25_N	260	1.7	0.1

**Table 9:** Capillary Water Absorption at 1440 min.

Specimen	Absorption (%)	Related to the Control (%)
R	4.4	100
NSP	3.5	79.7
SPN	3.0	68.5
NR	3.3	74.7
A_6_N	2.4	55.5
D_25_N	2.5	56.8

## Data Availability

The original contributions presented in this study are included in the article. Further inquiries can be directed to the corresponding author.

## Abbreviations

The following abbreviations are used in this manuscript:

OPC	Ordinary Portland cement
MICP	Microbial induced calcium carbonate precipitation
GBFS	Ground granulated blast furnace slag
CWP	Ceramic waste powder
NaOH	Sodium hydroxide
K_2_SiO_3_	Potassium silicate
SP	*Sporosarcina pasteurii*
A_6_	*Viridibacillus arenosi*
D_25_	*Bacillus zhangzhouensis*
ATCC	Medium 1376
½TSB	Tryptic Soy Broth
UPV	Ultrasonic Pulse Velocity
SEM-EDS	Scanning electron microscopy and energy-dispersive X-ray spectroscopy
XRD	X-ray diffraction
XRF	X-ray fluorescence
